# Activity Exerted by a Testosterone Derivative on Myocardial Injury Using an Ischemia/Reperfusion Model

**DOI:** 10.1155/2014/217865

**Published:** 2014-04-16

**Authors:** Figueroa-Valverde Lauro, Díaz-Cedillo Francisco, García-Cervera Elodia, Pool-Gómez Eduardo, López-Ramos Maria, Rosas-Nexticapa Marcela, Hau-Heredia Lenin, Sarabia-Alcocer Betty, Velázquez-Sarabia Betty Monica

**Affiliations:** ^1^Laboratory of Pharmaco-Chemistry, Faculty of Chemical Biological Sciences, University Autonomous of Campeche, Avenida Agustín Melgar s/n, Colonia Buenavista, 24039 San Francisco de Campeche, CAM, Mexico; ^2^Escuela Nacional de Ciencias Biológicas del Instituto Politéecnico Nacional, Prolongación de Carpio y Plan de Ayala s/n, Col. Santo Tomas, 11340 Mexico City, DF, Mexico; ^3^Facultad de Nutrición, Médicos y Odontologos s/n, Unidad del Bosque, 91010 Xalapa, VER, Mexico; ^4^Faculty of Medicine, University Autonomous of Campeche, Avenida Patricio Trueba de Regil s/n, Col Lindavista, 24090 San Francisco de Campeche, CAM, Mexico

## Abstract

Some reports indicate that several steroid derivatives have activity at cardiovascular level; nevertheless, there is scarce information about the activity exerted by the testosterone derivatives on cardiac injury caused by ischemia/reperfusion (I/R). Analyzing these data, in this study, a new testosterone derivative was synthetized with the objective of evaluating its effect on myocardial injury using an ischemia/reperfusion model. In addition, perfusion pressure and coronary resistance were evaluated in isolated rat hearts using the Langendorff technique. Additionally, molecular mechanism involved in the activity exerted by the testosterone derivative on perfusion pressure and coronary resistance was evaluated by measuring left ventricular pressure in the absence or presence of the following compounds: flutamide, prazosin, metoprolol, nifedipine, indomethacin, and PINANE TXA_2_. The results showed that the testosterone derivative significantly increases (*P* = 0.05) the perfusion pressure and coronary resistance in isolated heart. Other data indicate that the testosterone derivative increases left ventricular pressure in a dose-dependent manner (0.001–100 nM); however, this phenomenon was significantly inhibited (*P* = 0.06) by indomethacin and PINANE-TXA_2_  (*P* = 0.05) at a dose of 1 nM. In conclusion, these data suggest that testosterone derivative induces changes in the left ventricular pressure levels through thromboxane receptor activation.

## 1. Introduction

Several reports indicate that myocardial infarction is a major cause of death and disability worldwide [1, 2]; this cardiovascular disease is due to cell death of cardiac myocytes caused by prolonged myocardial ischemia. Acute myocardial infarction can produce alterations in the topography of both the infarcted and noninfarcted regions of the ventricle [3]. There are some reports which show that the most effective method of limiting necrosis is restoration of blood flow; however, the effects of reperfusion itself may also be associated with tissue injury [4]. A study [5] showed that ischemic preconditioning (PC) upregulates the expression and activity of COX-2 in the heart and that this increase in COX-2 activity mediates the protective effects of the late phase of PC against both myocardial stunning and myocardial infarction. Another study [6] indicates that activation of COX-2 produces cardioprotection via synthesis or release of prostanoids such as PGI_2_ and PGE_2_ which induce a cardiac protective effect against ischemia-reperfusion injury in experimental animals [7–9]. It is important to mention that there are studies which suggest that the release of these prostanoids may be the result of the effect induced by some steroids on the COX-2 activity; this phenomenon results in a decrease in the ischemia-reperfusion injury [10–13]. For example, there is a study which shows that 17*β*-estradiol reduced injury by ischemia/reperfusion via activation of PGI_2_ in an animal model [14]. However, another data showed that administration of other types of steroid (testosterone) is associated with a reduced susceptibility to myocardial ischemia and this phenomenon results in the regulation of intracellular calcium levels on ischemia/reperfusion injury [15]. In addition, another study shows that testosterone exerted activation of STAT3 (signal transducers and activators of transcription 3) and SOCS3 (suppressor of cytokine signaling 3) after ischemia/reperfusion injury [16]. Another report indicates that testosterone indirectly regulates the activation of Akt (protein kinase) during cardiac ischemia/reperfusion [17]. However, other reports suggest that endogenous testosterone may have a negative effect on the heart subjected to acute ischemia/reperfusion via androgen receptor [18, 19]. Furthermore, a study [20] demonstrated that administration of a derivative of testosterone (nandrolone) increases ischemia/reperfusion by changes in the concentrations of both AMP (adenosine monophosphate) and TNF*α* (tumor necrosis factor alpha). Another data indicate that another type of testosterone derivative (dehydroepiandrosterone) regulates gene expression of both VEGF (vascular endothelial growth factor) and interleukins (IL-1 and IL-6) on ischaemia/reperfusion injury [21]. All these experimental results indicate that testosterone and its derivatives exert effects on ischemia/reperfusion injury; however, the cellular site and actual molecular mechanisms of testosterone and its derivatives are very confusing; therefore, data are needed for characterizing the activity induced by this steroid and its derivative on ischemia-reperfusion injury. To provide this information, the present study was designed to investigate the effects of testosterone and its derivative in an ischemia/reperfusion injury model. In addition, the activity of testosterone and its derivative on perfusion pressure and coronary resistance were evaluated in isolated rat hearts using the Langendorff technique. In order to evaluate the molecular mechanism involved in the activity induced by the testosterone derivative on left ventricular pressure the following compounds were used as pharmacological tools: flutamide (androgenic receptor antagonist) [22], prazosin (*α*_1_ adrenoreceptor antagonist) [23], metoprolol (selective *β*_1_ receptor blocker) [24], nifedipine (antagonist of calcium-channel) [25], indomethacin (a nonselective inhibitor of cyclooxygenase) [26], and PINANE TXA_2_ (thromboxane receptor antagonist) [27].

## 2. Material and Methods

### 2.1. Chemical Synthesis

The compounds evaluated in this study were purchased from Sigma-Aldrich Co., Ltd. The melting point for the testosterone derivative was determined with an Electrothermal (900 model). Infrared spectra (IR) were recorded using KBr pellets on a Perkin Elmer Lambda 40 spectrometer. ^1^H and ^13^C NMR (nuclear magnetic resonance) spectra were recorded on a Varian VXR-300/5 FT NMR spectrometer at 300 and 75.4 MHz (megahertz) in CDCl_3_ (deuterated chloform) using TMS (tetramethylsilane) as internal standard. EIMS (electron impact mass spectroscopy) spectra were obtained with a Finnigan Trace Gas Chromatography Polaris Q Spectrometer. Elementary analysis data were acquired from a Perkin Elmer Ser. II CHNS/0 2400 elemental analyzer.

#### 2.1.1. Synthesis of 1-[4-(2-Amino-ethylimino)-4-(4-fluoro-cyclohexyl)-butyl]-4-(4-chloro-phenyl)-piperidin-4-ol (Compound** 3**)

A solution of haloperidol (100 mg, 0.26 mmol), ethylenediamine (80 *μ*L, 1.19 mmol), and boric acid (40 mg, 0.65 mmol) in 10 mL of methanol was stirred for 72 h at room temperature. The reaction mixture was evaporated to dryness under reduced pressure; the residue washed 4 times with water. Then the precipitate was separated and dried at room temperature.

#### 2.1.2. Synthesis of 4-(4-Chloro-phenyl)-1-{4-(4-fluoro-phenyl)-4-[2-(17-hydroxy-10,13-dimethyl-1,2,6,7,8,9,10,11,12,13,14,15,16,17-tetradecahydro-cyclopenta[a]phenanthren-3-ylideneamino)-ethylimino]-butyl}-piperidin-4-ol (Compound** 5**)

A solution of compound** 3** (100 mg, 0.24 mmol), testosterone (70 mg, 0.24 mmol), and boric acid (40 mg, 0.65 mmol) in 10 mL of methanol was stirred for 72 h at room temperature. The reaction mixture was evaporated to dryness under reduced pressure; the residue washed 3 times with water. Then the precipitate was separated and dried at room temperature.

### 2.2. Biological Method

All experimental procedures and protocols used in this investigation were reviewed and approved by the Animal care and use Committee of University Autonomous of Campeche (no. PI-420/12) and were in accordance with the Guide for the Care and Use of Laboratory Animals [28]. Male Wistar rats (*n* = 105), weighing 200–250 g, were obtained from University Autonomous of Campeche.

### 2.3. Reagents

All drugs were dissolved in methanol and different dilutions were obtained using Krebs-Henseleit solution (≤0.01%, v/v).

### 2.4. Experimental Design

Briefly, the male rat (200–250 g) was anesthetized by injecting them with pentobarbital at a dose rate of 50 mg/Kg body weight. Then the chest was opened, and a loose ligature passed through the ascending aorta. The heart was then rapidly removed and immersed in ice cold physiologic saline solution. The heart was trimmed of noncardiac tissue and retrograde perfused via a noncirculating perfusion system at a constant flow rate. The perfusion medium was the Krebs-Henseleit solution (pH = 7.4, 37°C) composed of (mmol) 117.8, NaCl; 6, KCl; 1.75, CaCl_2_; 1.2, NaHPO_4_; 1.2, MgSO_4_; 24.2, NaHCO_3_; 5, glucose; 7 and 5, sodium pyruvate. The solution was actively bubbled with a mixture of O_2_/CO_2_ (95 : 5/5%). The coronary flow was adjusted with a variable speed peristaltic pump. An initial perfusion rate of 15 mL/min for 5 min was followed by a 15 min equilibration period at a perfusion rate of 10 mL/min. All experimental measurements were done after this equilibration period.

### 2.5. Perfusion Pressure

Evaluation of measurements of perfusion pressure changes induced by drugs administration in this study was assessed using a pressure transducer connected to the chamber where the hearts were mounted and the results entered into a computerized data capture system (Biopac).

### 2.6. Inotropic Activity

Contractile function was assessed by measuring left ventricular developed pressure (LV/dP), using a saline-filled latex balloon (0.01 mm, diameter) inserted into the left ventricle via the left atrium. The latex balloon was bound to cannula which was linked to pressure transducer that was connected with the MP100 data acquisition system.

#### 2.6.1. First Stage

*Activity Induced by Testosterone and Its Derivative Using an Ischemia/Reperfusion Model.* After 15 minutes of equilibration time, the hearts were subjected to ischemia for 30 minutes by turning off the perfusion system [29]. After this period, the system was restarted and the hearts were reperfused by 30 minutes with Krebs-Henseleit solution. The hearts were randomly divided into 3 major treatment groups with *n* = 9.

 
*Group  I.* Hearts were subjected to ischemia/reperfusion but received vehicle only (Krebs-Henseleit solution).
 
*Group  II.* Hearts were subjected to ischemia/reperfusion and treated with testosterone at a dose of 0.001 nM before ischemia period (for 10 minutes) and during the entire period of reperfusion.
 
*Group  III.* Hearts were subjected to ischemia/reperfusion and treated with the testosterone derivative at a dose of 0.001 nM before ischemia period (for 10 minutes) and during the entire period of reperfusion.

It is noteworthy that, at the end of each experiment, the perfusion pump was stopped and 0.5 mL of fluorescein solution (0.10%) was injected slowly through a sidearm port connected to the aortic cannula. The dye was passed through the heart for 10 sec to ensure its uniform tissue distribution. The presence of fluorescein was used to demarcate the tissue that was not subjected to regional ischemia, as opposed to the risk region. The heart was removed from the perfusion apparatus and cut into two transverse sections at right angles to the vertical axis. The right ventricle, apex, and atrial tissue were discarded. The areas of the normal left ventricle nonrisk region, area at risk, and infarct region were determined using the technique reported by both and coworkers [29]. Total area at risk was expressed as the percentage of the left ventricle.

#### 2.6.2. Second Stage

*Effect Induced by Testosterone and Its Derivative on Perfusion Pressure.* Changes in perfusion pressure as a consequence of increases in time (3 to 18 min) in the absence (control) or presence of testosterone and its derivative at a concentration of 0.001 nM were determined. The effects were obtained in isolated hearts perfused at a constant flow rate of 10 mL/min.

*Evaluation of Effects Exerted by the Testosterone and Its Derivative on Coronary Resistance. *The coronary resistance in the absence (control) or presence of testosterone and its derivative at a concentration of 0.001 nM was evaluated. It is noteworthy that Coronary resistance was calculated as the ratio of perfusión pressure at coronary flow assayed (10 mL/min).

#### 2.6.3. Third Stage

*Effects Induced by Testosterone and Its Derivative on Left Ventricular Pressure through Androgen Receptors*. Intracoronary boluses (50 *μ*L) of testosterone and its derivative (0.001 to 100 nM) were administered and the corresponding effect on the left ventricular pressure was determined. The dose-response curve (control) was repeated in the presence of flutamide at a concentration of 1 nM (duration of preincubation with flutamide was by a 10 min equilibration period).

*Effects Induced by the Testosterone Derivative on Left Ventricular Pressure through β*_1_ *-Adrenergic Receptor*. Intracoronary boluses (50 *μ*L) of the testosterone derivative (0.001 to 100 nM) were administered and the corresponding effect on the left ventricular pressure was determined. The dose-response curve (control) was repeated in the presence of metoprolol at a concentration of 1 nM (duration of preincubation with metoprolol was by a 10 min equilibration period).

*Effects Exerted by the Testosterone Derivative on Left Ventricular Pressure through α*_1_ *-Adrenergic Receptor*. Intracoronary boluses (50 *μ*L) of the testosterone derivative (0.001 to 100 nM) were administered and the corresponding effect on the left ventricular pressure was determined. The dose-response curve (control) was repeated in the presence of prazosin at a concentration of 1 nM (duration of preincubation with prazosin was by a 10 min equilibration period).

*Effects of the Testosterone Derivative on Left Ventricular Pressure through the Calcium Channel*. Intracoronary boluses (50 *μ*L) of the testosterone derivative [0.001 to 100 nM] were administered and the corresponding effect on the left ventricular pressure was evaluated. The dose-response curve (control) was repeated in the presence of nifedipine at a concentration of 1 nM (duration of the preincubation with nifedipine was for a period of 10 min).

*Effect Exerted by the Testosterone Derivative on Left Ventricular Pressure in the Presence of Indomethacin.* The boluses (50 *μ*L) of the testosterone derivative [0.001 to 100 nM] were administered and the corresponding effect on the left ventricular pressure was evaluated. The bolus injection administered was done in the point of cannulation. The dose-response curve (control) was repeated in the presence of indomethacin at a concentration of 1 nM (duration of the preincubation with indomethacin was for a period of 10 min).

*Effects of the Testosterone Derivative on Left Ventricular Pressure through the TXA*_*2*_ *Receptor Activation*. Intracoronary boluses (50 *μ*L) of the testosterone derivative [0.001 to 100 nM] were administered and the corresponding effect on the left ventricular pressure was evaluated. The dose-response curve (control) was repeated in the presence of PINANE TXA_2_ at a concentration of 1 nM (duration of the preincubation with PINANE TXA_2_ was for a period of 10 min).

#### 2.6.4. Fourth Stage

*Activity Induced by Indomethacin and PINANE TXA*_*2*_ *in Presence y Absence of the Testosterone Derivative Using an Ischemia/Reperfusion Model.* After 15 minutes of equilibration time, the hearts were subjected to ischemia for 30 minutes by turning off the perfusion system [29]. After this period, the system was restarted and the hearts were reperfused by 30 minutes with Krebs-Henseleit solution. The hearts were randomly divided into 3 major treatment groups with *n* = 6.

 
*Group  I.* Hearts were subjected to ischemia/reperfusion but received vehicle only (Krebs-Henseleit solution).
 
*Group  II.* Hearts were subjected to ischemia/reperfusion and treated with the testosterone derivative at a dose of 0.001 nM before ischemia period (for 10 minutes) and during the entire period of reperfusion.
 
*Group  III.* Hearts were subjected to ischemia/reperfusion and treated with testosterone derivative at a dose of 0.001 nM before ischemia period (for 10 minutes) and during the entire period of reperfusion. The dose-response curve (control) was repeated in the presence of indomethacin at a concentration of 1 nM.
 
*Group  IV.* Hearts were subjected to ischemia/reperfusion and treated with testosterone derivative at a dose of 0.001 nM before ischemia period (for 10 minutes) and during the entire period of reperfusion. The dose-response curve (control) was repeated in the presence of PINANE TXA_2_ at a concentration of 1 nM.
 
*Group  V.* Hearts were subjected to ischemia/reperfusion and treated with the indomethacin at a dose of 0.001 nM before ischemia period (for 10 minutes) and during the entire period of reperfusion.
 
*Group  VI.* Hearts were subjected to ischemia/reperfusion and treated with the PINANE TXA_2_ at a dose of 0.001 nM before ischemia period (for 10 minutes) and during the entire period of reperfusion.

The areas of the normal left ventricle nonrisk region, area at risk, and infarct region were determined using the technique reported by both and coworkers [29]. Total area at risk was expressed as the percentage of the left ventricle.

### 2.7. Statistical Analysis

The obtained values are expressed as average ± SE. The data obtained were put under analysis of variance (ANOVA) with the Bonferroni correction factor using the SPSS 12.0 program [30]. The differences were considered significant when *P* was equal or smaller than 0.05.

## 3. Results

**Figure 1:**
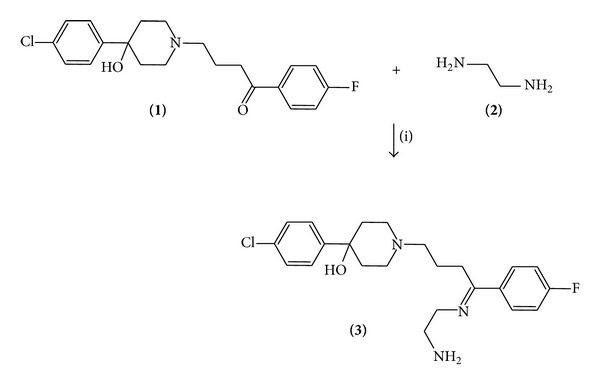
Synthesis of 1-[4-(2-amino-ethylimino)-4-(4-fluoro-cyclohexyl)-butyl]-4-(4-chloro-phenyl)-piperidin-4-ol (**3**). Reaction of haloperidol with ethylenediamine using boric acid as catalyst (i) to form the compound** 3**.

The yielding of compound** 3 **(Figure 1) was of 85% with melting point of 78–80°C. In addition, the spectroscopic analyses showed signals for IR (*V*_max⁡_, cm^−1^) at 3412, 3382, and 3330. The chemical shifts of the spectroscopic analyses of ^1^H NMR and ^13^C NMR for compound** 3 **are showed in Tables 1 and 2. Finally, the results of mass spectroscopy (MS) (70 eV) are shown, *m*/*z* 417.10 [M^+^, 10]. Additionally, the elementary analysis data for compound** 3 **(C_23_H_29_ClFN_3_O) was calculated (C, 66.10; H, 6.99; Cl, 8.48; F, 4.55; N, 10.05; O, 3.83) and found (C, 66.08; H, 6.97).

**Figure 2:**
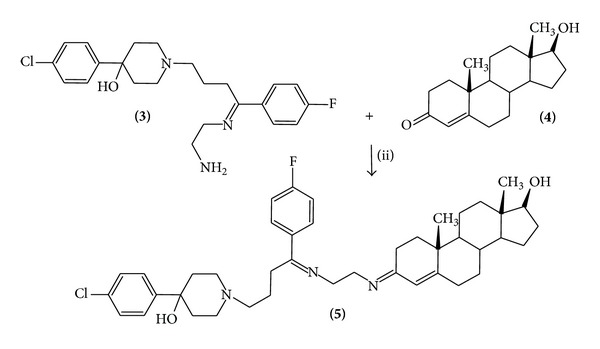
Synthesis of 4-(4-chloro-phenyl)-1-{4-(4-fluoro-phenyl)-4-[2-(17-hydroxy-10,13-dimethyl-1,2,6,7,8,9,10,11,12,13,14,15,16,17-tetradecahydro-cyclopenta[a]phenan thren-3-ylideneamino)-ethylimino]-butyl}-piperidin-4-ol (**5**). Reaction of 1-[4-(2-amino-ethylimino)-4-(4-fluoro-cyclohexyl)-butyl]-4-(chloro-phenyl)-piperidin-4-ol (**3**) with testosterone (**2**) to form the compound** 3**. (ii) = boric acid.

Furthermore, other results (Figure 2) showed a yielding of 64% and a melting point of 170–172°C, for the testosterone derivative (compound** 5**). Additionally, the spectroscopic analyses showed signals for IR (*V*_max⁡_, cm^−1^) at 3410, 3,338, and 2912. The chemical shifts of the spectroscopic analyses of ^1^H NMR and ^13^C NMR for the testosterone derivative are showed in Tables 3 and 4. Finally, the results of mass spectroscopy (MS) (70 eV) are shown, *m*/*z* 687.32 [M^+^,12]. Additionally, the elementary analysis data for compound** 5 **(C_42_H_55_ClFN_3_O_2_) was calculated (C, 73.28; H, 8.05; Cl, 5.15; F, 2.76; N, 6.10; O, 4.65) and found (C, 73.26; H, 8.04).

### 3.1. Biological Evaluation

#### 3.1.1. First Stage

**Figure 3:**
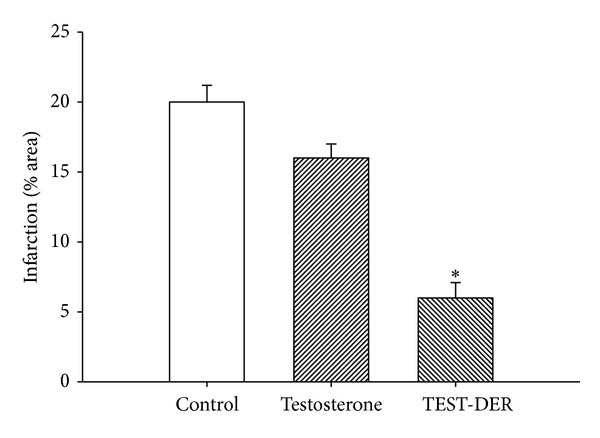
Effect exerted by the testosterone and its derivative (TEST-DER) on cardiac ischemia/reperfusion. The results showed that the testosterone derivative significantly reduced infarct size expressed as a percentage of the area at risk compared with testosterone and the vehicle-treated hearts (**P** = 0.06). Each bar represents the mean ± SE of 9 experiments.

*Activity Induced the Testosterone Derivative Using an Ischemia/Reperfusion Model.* The results (Figure 3) showed that the testosterone derivative significantly reduced (*P* = 0.06) infarct size (expressed as a percentage of the area at risk) compared with both testosterone and vehicle-treated hearts.

#### 3.1.2. Second Stage

**Figure 4:**
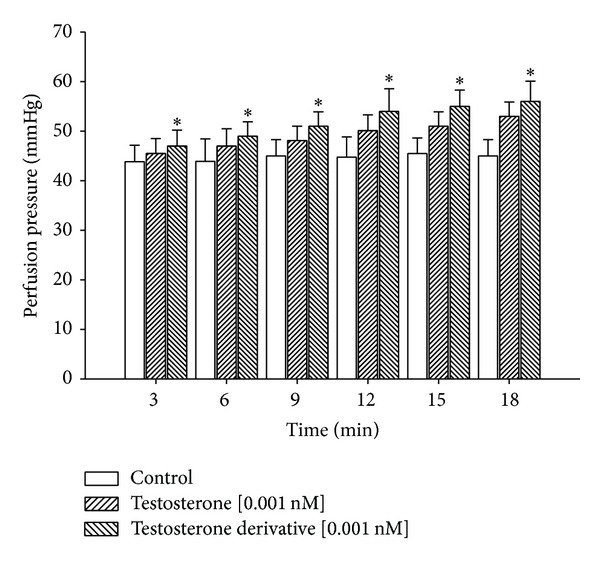
Effect induced by the testosterone and its derivative on perfusion pressure. The results showed that the testosterone derivative significantly increases perfusion pressure (**P** = 0.05) through time in comparison with the control conditions and testosterone. Each bar represents the mean ± SE of 9 experiments.

**Figure 5:**
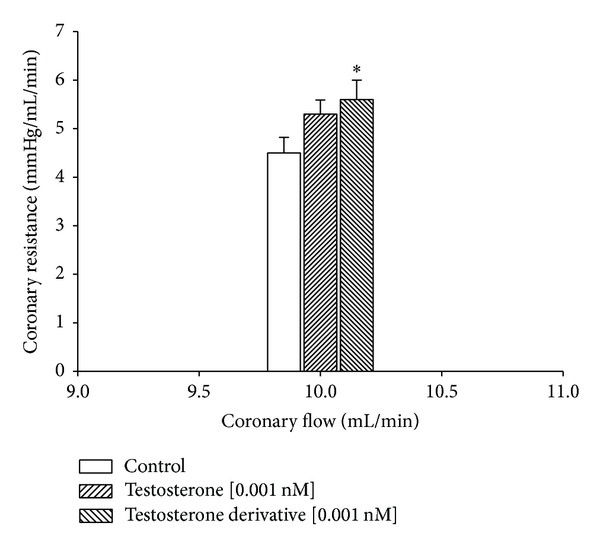
Activity exerted by the testosterone derivative and its derivative on coronary resistance. The results show that coronary resistance was higher (**P** = 0.05) in the presence of the testosterone derivative in comparison with the control conditions and testosterone. Each bar represents the mean ± SE of 9 experiments.

In this study, the activity induced by the testosterone and its derivative on perfusion pressure and coronary resistance in the isolated rat heart was evaluated. The results obtained (Figure 4) from changes in perfusion pressure as a consequence of increases in the time (3–18 min) showed that the testosterone derivative at a dose of 0.001 nM significantly increases the perfusion pressure (**P** = 0.05) in comparison with the control conditions and testosterone [0.001 nM]. Other result (Figure 5) showed that coronary resistance, calculated as the ratio of perfusion pressure at coronary flow assayed (10 mL/min), was significantly higher (**P** = 0.05) in the presence of steroid derivative [0.001 nM] than in control conditions and testosterone [0.001 nM].

#### 3.1.3. Third Stage

**Figure 6:**
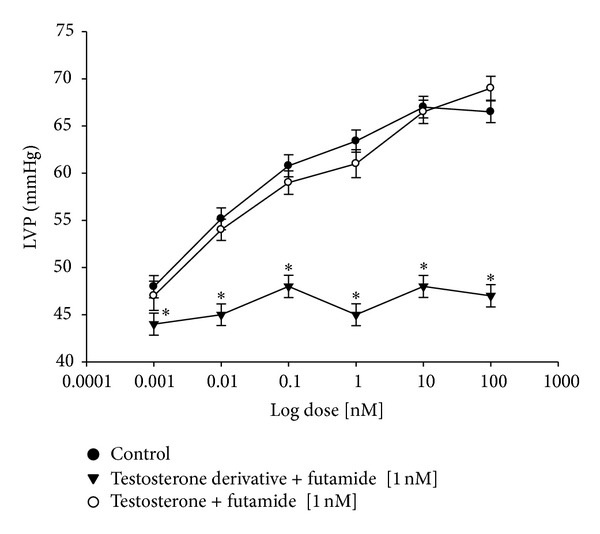
Effects induced by the testosterone and its derivative on LVP through androgen receptor. Intracoronary boluses (50 *μ*L) of the testosterone and its derivative [0.001 to 100 nM] were administered and the corresponding effect on the LVP was determined. The dose-response curve (control) was repeated in the presence of flutamide (duration of preincubation with flutamide was by a 10 min equilibration period). The results showed that only the activity exerted of testosterone on LVP was significantly inhibited (**P** = 0.06). Each bar represents the mean ± SE of 9 experiments. LVP: left ventricular pressure.

Figure 6 showed that the testosterone and its derivative induce an increase on left ventricular pressure in a dose-dependent manner [0.001 to 100 nM]; however, only the effect exerted by testosterone was significantly inhibited by flutamide at a dose of 1 nM (**P** = 0.06).

**Figure 7:**
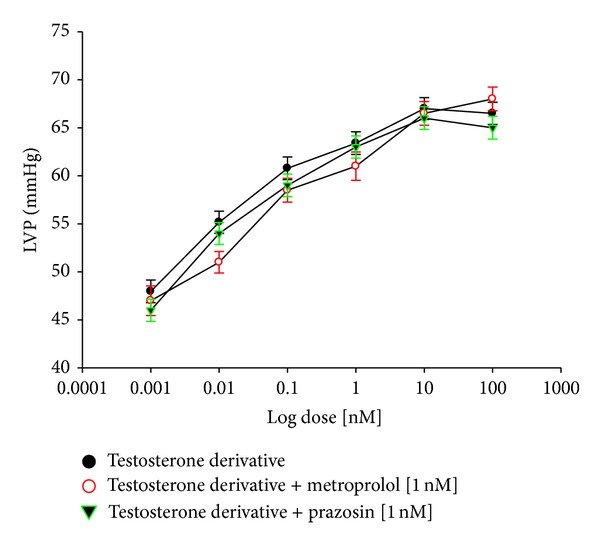
Activity exerted by the testosterone derivative on LVP through of adrenergic receptors. Testosterone derivative [0.001 to 100 nM] was administered (intracoronary boluses, 50 *μ*L) and the corresponding effect on the LVP was evaluated in the absence and presence of prazosin or metoprolol. The results showed that activity induced by the testosterone derivative on LVP was not inhibited in the presence of prazosin or metoprolol. Each bar represents the mean ± SE of 9 experiments. LVP: left ventricular pressure.

**Figure 8:**
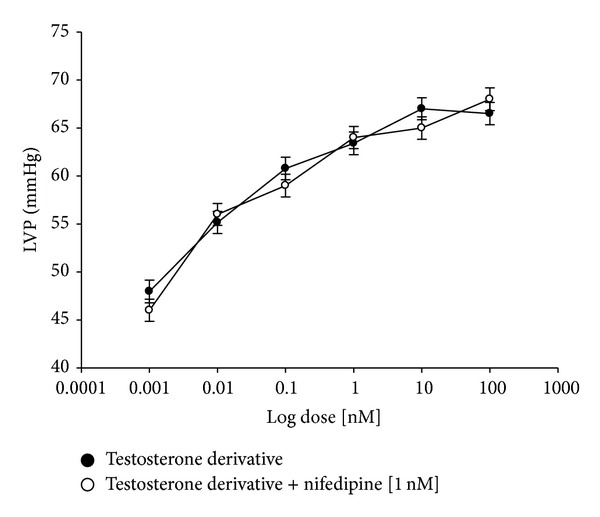
Effects induced by the testosterone derivative on LVP through calcium channel activation. Intracoronary boluses (50 *μ*L) of the testosterone derivative [0.001 to 100 nM] were administered and the corresponding effect on the LVP was determined in the absence and presence of nifedipine. The results showed that the testosterone derivative increases the LVP in a dose-dependent manner and this effect was not inhibited in the presence of nifedipine. Each bar represents the mean ± SE of 9 experiments. LVP: left ventricular pressure.

**Figure 9:**
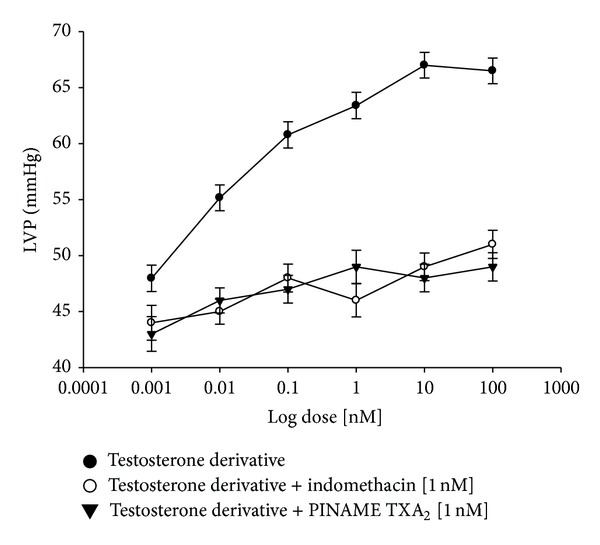
Effects induced by the testosterone derivative on LVP through prostaglandins synthesis and thromboxane receptor. Intracoronary boluses (50 *μ*L) of the testosterone derivative [0.001 to 100 nM] were administered and the corresponding effect on the LVP was determined in the absence and presence of indomethacin and PINANE TXA_2_. The results showed that the testosterone derivative increases the LVP in a dose-dependent manner and this effect was significantly inhibited in the presence of indomethacin (*P* = 0.05) and PINANE TXA_2_ (*P* = 0.05). Each bar represents the mean ± SE of 9 experiments. LVP: left ventricular pressure.

On the other hand, other experiments showed that the testosterone derivative increased left ventricular pressure in a dose-dependent manner [0.001 to 100 nM] and this effect was not inhibited in presence of prazosin or metoprolol (Figure 7) at a concentration of 1 nM. Additionally, other results indicate that effect induced by the testosterone derivative on left ventricular pressure (Figure 8) in presence of nifedipine at a concentration of 1 nM was not blocked. Finally, other results (Figure 9) indicate that activity exerted by the testosterone derivative [0.001 to 100 nM] on left ventricular of testosterone was significantly blocked in presence of indomethacin (**P** = 0.05) and PINAME TXA_2_ (**P** = 0.05) at a dose of 1 nM.

#### 3.1.4. Fourth Stage

**Figure 10:**
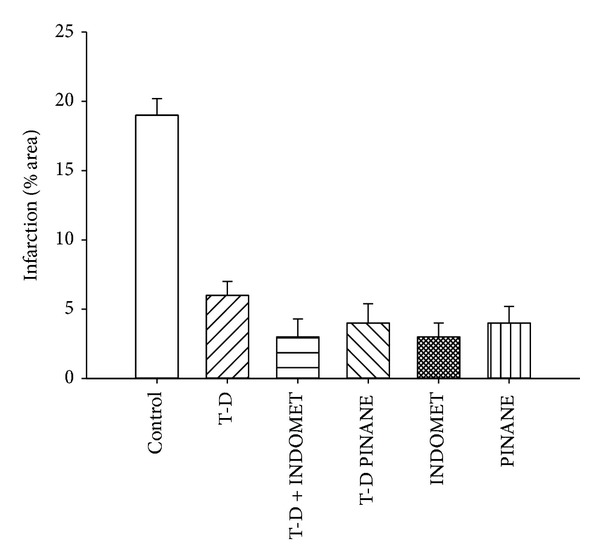
Effect exerted by indomethacin (INDOMET) and PINANE TXA2 (PINANE) in presence and absence of testosterone derivative (T-D) on cardiac ischemia/reperfusion.The results showed that T-D significantly reduced infarct size expressed as a percentage of the area at risk compared with testosterone and the vehicle-treated hearts (**P** = 0.06). However, this effect was partially blocked by INDOMET and PINANE. Other data indicate that INDOMET significantly decreased infarct size (*P* = 0.05) in comparison with PINANE. Each bar represents the mean ± SE of 6 experiments.

The results (Figure 10) showed that the testosterone derivative significantly reduced (*P* = 0.06) infarct size (expressed as a percentage of the area at risk) compared with vehicle-treated hearts. However, this effect was partially blocked by indomethacin and PINANE TXA_2_. Other data indicate that Indomethacin significantly decreased infarct size (*P* = 0.05) in comparison with testosterone derivative, PINANE TXA_2_, and vehicle-treated hearts.

## 4. Discussion

### 4.1. Chemical Synthesis

There are many procedures for preparation of several androgen derivatives; nevertheless, despite its wide scope, have some drawbacks; for example, several agents used have limited stability and their preparation requires special conditions [31, 32]. Analyzing these data, we report a straightforward route for synthesis of an androgen derivative using some strategies. The first stage was achieved by the synthesis of 1-[4-(2-amino-ethylimino)-4-(4-fluoro-cyclohexyl)-butyl]-4-(4-chloro-phenyl)-piperidin-4-ol (**3**) which has an imine group (Schiff base) involved in their chemical structure (Figure 1). It is noteworthy that there are several procedures for the synthesis of imine groups which are described in the literature [33, 34]. However, in this study, the synthesis of compound** 3** was developed by the reaction of haloperidol with ethylenediamine using boric acid as catalyst to form** 3**. The structure of compound** 3** was confirmed using IR and NMR spectroscopy (Tables 1 and 2). The IR spectra contained characteristic vibrations at 3412 for hydroxyl group; at 3382 for amino group; at 3330 for imino groups, and 2910 piperidine ring. The ^1^H NMR spectrum of** 3** shows signals at 1.52–1.69 ppm for piperidine ring; at 1.71–2.50 ppm for methylene groups bound to both imino and piperidine groups; at 2.75–3.03 ppm for piperidine ring; at 3.08–3.51 ppm for methylene groups bound to both imino and amino groups; at 4.63 ppm for both hydroxyl and amino groups; at 7.06–8.10 ppm for both phenyl groups. The ^13^C NMR spectra showed chemical shifts at 26.15, 28.47, and 53.63 ppm for methylene groups bound to both imino and piperidine groups; at 38.40, 47.04, and 70.12 ppm for piperidine ring; at 41.02 and 54.09 ppm for methylene groups bound to both amino and imino groups; at 115.08–145.22 and 163.30 ppm for both phenyl groups; at 162.11 ppm for imino group. Finally, the presence of testosterone derivative was further confirmed from mass spectrum which showed a molecular ion at *m*/*z* 417.12.

The second stage was achieved by the synthesis of the testosterone derivative by the reaction of compound** 3** with testosterone using boric acid as catalyst to form a new imino group involved in the chemical structure of the steroid derivative. The structure of the testosterone derivative was confirmed using IR and NMR spectroscopy (Tables 3 and 4). The IR spectra contained characteristic vibrations at 3410 for hydroxyl group; at 3338 for both imino groups and 2912 piperidine ring. The ^1^H NMR spectrum of the testosterone derivative shows signals at 0.80 and 1.05 ppm for methyl groups bound to steroid nucleus; at 0.96–1.02, 1.08–1.48, 1.58–1.62, 1.69, 1.86–2.38, 3.60, and 5.86 ppm for steroid nucleus; at 1.54, 1.64 and 2.78–3.00 ppm for piperidine ring; at 1.74 and 2.42–2.54 ppm for methylene groups bound to both piperidine ring and imine groups; at 3.70 and 3.80 3.99 ppm for methylene groups bound to both imino groups. Finally, two signals at 4.74 ppm for both hydroxyl groups and at 6.90–8.10 ppm for phenyl groups were found.

On the other hand, the ^13^C NMR spectra showed chemical shifts at 11.10 and 17.70 ppm for both methyl groups bound to steroid nucleus; at 21.08–23.32, 30.26–30.66, 42.80, 50.20–50.56, 80.78, 115.38, and 157.40 ppm for steroid nucleus; at 26.78–28.72 and 54.22 ppm for methylene groups bound to both piperidine and imino groups; at 38.30, 47.04, and 70.00 ppm for piperidine ring; at 51.70–52.40 ppm for methylene groups bound to both imino groups; at 115.06, 126.80, 136.72, and 163.84 ppm for phenyl group bound to imino group; at 128.60–136.17 and 145.18 ppm for phenyl group bound to piperidine ring; at 162.12 and 165.82 ppm for both imino groups. Finally, the presence of testosterone derivative was further confirmed from mass spectrum which showed a molecular ion at *m*/*z* 687.36.

### 4.2. Biological Evaluation

In this study, the activity of the testosterone derivative on myocardial injury using an ischaemia/reperfusion model was evaluated. The results showed that the testosterone derivative significantly reduced infarct size (expressed as a percentage of the area at risk) compared with both testosterone and vehicle-treated hearts. This effect can be conditioned by changes in the chemical structure of testosterone which may consequently bring activation of some biological structures (e.g., ionic channels or specific receptors) involved in the endothelium of coronary artery [35] or by the influence exerted by the testosterone derivative on blood pressure which consequently bring reduction in the infarct size and decrease the myocardial injury after ischemia/reperfusion similar to other reports for other types of steroids [36]. To assess these hypotheses, the effect induced by the testosterone and its derivative on blood vessel capacity and coronary resistance, translated as changes in perfusion pressure, was evaluated in an isolated rat heart model. The experimental results showed that the testosterone derivative significantly increases the perfusion pressure over time (3–18 min) compared with testosterone and the control conditions. These data suggest that activity induced by the testosterone derivative on perfusion pressure could modify vascular tone and coronary resistance of heart. Therefore, in this study, the activity exerted by testosterone and its derivative on coronary resistance was evaluated. The results indicate that coronary resistance was increased in presence of the steroid derivative. These data suggest that the testosterone derivative exerts effects on vascular tone through the generation or activation of vasoactive substances such as intracellular calcium. This phenomenon is similar to the activity exerted by other compounds such as the carbamazepine-alkyne derivative [37].

In order to characterize the molecular mechanism of this phenomenon and analyze the reports of some investigations which indicate that testosterone induces its effect on blood pressure via activation of the androgen receptor [38], we used flutamide (androgen-receptor blocker) to determine if the effects of testosterone and its derivative on perfusion pressure were via the androgen receptor. Our results showed that only the effect of testosterone was significantly inhibited in presence of flutamide; however, the testosterone derivative was not inhibited by the androgen-receptor blocker, suggesting that the molecular mechanism for the testosterone derivative is not via the androgenic receptor. In search of molecular mechanism involved in the activity of the testosterone derivative and analyzing a study which indicates that some androgens such as dehydroisoandrosterone 3-sulfate stimulate catecholamines production and this phenomenon may induce a positive inotropic effect [39, 40] which has an important role in the development or maintenance of elevated blood pressure [41]. For this reason, in this study, the effect exerted by the testosterone derivative on left ventricular pressure was evaluated in the absence or presence of prazosin or metoprolol. The experimental results showed that the effect induced by the testosterone derivative was not inhibited in the presence of these compounds. These data indicated that the molecular mechanism involved in the activity of testosterone derivative is not via adrenergic system.

Analyzing the results obtained and other reports which indicate that some steroid derivatives can induce changes on blood pressure by increasing calcium levels [42]; in this study, we also considered validating the effect induced by the testosterone derivative on left ventricular pressure via the calcium channels activation using as pharmacological tool to nifedipine. The results showed that the effect induced by the testosterone derivative was not inhibited in the presence of nifedipine. These results indicate that activity of the testosterone derivative was not through calcium channels activation.

On the other hand, in the search of the molecular mechanism involved in effect induced by the testosterone derivative on left ventricular pressure and analyzing previous reports, which indicate that some steroids exert its effect on left ventricular pressure via prostaglandins synthesis [43]. In this sense, other alternative experiments were conducted to evaluate the possibility that the activities exerted by the testosterone derivative on left ventricular pressure involve stimulation and secretion of prostaglandins using as pharmacological tool indomethacin. The results showed that effect induced by the testosterone derivative on left ventricular pressure was blocked by indomethacin. These results indicate that the molecular mechanism involved in the effect exerted by the testosterone derivative was via prostaglandins. To assess whether the activity exercised by the derivative of testosterone on left ventricular pressure involves the activation or release of a specific prostaglandin such as thromboxane A_2_ as (TXA_2_) which is a substance that induces a vasoconstriction effect in heart [44]. In addition, it is important to mention that TXA_2_ has been found into the coronary circulation in patients with ischemic heart disease [45]. Therefore, in this study, other experiments were conducted using thromboxane receptor antagonist (PINANE TXA_2_) as pharmacological tool. The results indicate that effect exerted by the testosterone derivative was significantly inhibited by PINANE TXA2; these experimental data suggest that activity of the testosterone derivative on left ventricular pressure could be through receptor thromboxane A2 activation which consequently brings decrease in the ischemia/reperfusion injury. To assess this hypothesis, several experiments were conducted to evaluate the activity of indomethacin and PINANE TXA_2_ on ischemia/reperfusion injury in the presence or absence of the testosterone derivative. The results showed that effect exerted by steroid derivative on ischemia/reperfusion injury was inhibited partiality with indomethacin and PINANE TXA_2_. Additionally, other data showed that beneficial effects on ischemia/reperfusion injury induced by indomethacin and PINANE TXA_2_ were higher in comparison with testosterone derivative. All these data indicate that; (1) indomethacin blocked the activity of COX and this phenomenon consequently bring inhibition of synthesis or release to two prostaglandins such as PGI2 (prostaciclin) and TXA2 (tromboxane A_2_) which exert effects on ischemia/reperfusion injury such as happening in other types of studies [7–9, 14]; (2) the testosterone derivative decreases the ischemia/reperfusion injury through thromboxane receptor activation; (3) the steroid derivative could induce an imbalance in the relation of PGI_2_/TXA_2_ which may consequently bring changes in the metabolism of myocardium and induce a decrease of the ischemia/reperfusion injury.

## 5. Conclusions

The testosterone derivative is a particularly interesting drug, because the activity induced for this compound on ischemia/reperfusion injury involves a molecular mechanism different in comparison with other drugs. This phenomenon may constitute a novel therapy for ischemia/reperfusion injury.

## Figures and Tables

**Table 1:** ^
1^H NMR (300 MHz, CDCl_3_) data for the haloperidol derivative (compound **3**).

1.52–1.69 (m, 4H), 1.71 (t, 2H, *J* = 6.9 Hz), 2.31 (t, 2H, *J* = 6.9 Hz), 2.50 (t, 2H, *J* = 6.9 Hz), 2.753.03 (m, 4H), 3.08 (t, 2H,*J* = 6.5 Hz,), 3.51 (t, 2H, *J* = 6.5 Hz), 4.63 (s, 3H), 7.06–8.10 (m, 8H) ppm.	

**Table 2:** ^
13^C NMR (300 MHz, CDCl_3_) data for the haloperidol derivative (compound **3**).

26.15, 28.47, 38.40, 41.02, 47.04, 53.63, 54.09, 70.12, 115.08, 126.80, 128.64, 129.15, 134.40, 136.17, 145.22, 162.11, 163.30 ppm.	

**Table 3:** ^
1^H NMR (300 MHz, CDCl_3_) data for the testosterone derivative (compound **5**).

0.80 (s, 3H), 0.96–1.02 (m, 3H), 1.05 (s, 3H), 1.08–1.48 (m, 6H), 1.54 (m, 2H), 1.58–1.62 (m, 3H), 1.64 (m, 2H), 1.69 (m, 1H), 1.74 (t, 2H, *J* = 6.54), 1.86–2.38 (m, 7H), 2.42 (t, 2H, J = 6.54), 2.54 (t, 2H, J = 6.54), 2.78–3.00 (m, 4H), 3.60 (m, 1H), 3.70 (t, 2H, *J* = 6.54), 3.80 (t, 2H, *J* = 6.54), 4.74 (broad, 2H), 5.86 (m, 1H), 6.90–8.10 (m, 8H) ppm.	

**Table 4:** ^
13^C NMR (300 MHz, CDCl_3_) data for the testosterone derivative (compound **5**).

11.10, 17.70, 21.08, 23.32, 26.78, 28.72, 30.26, 30.38, 31.10, 31.28, 31.70, 35.20, 35.34, 36.66, 38.30, 42.80, 47.04, 50.20, 50.56, 51.70, 52.40, 54.22, 70.00, 80.78, 115.08, 115.38, 126.80, 128.60, 129.15, 136.17, 136.72, 145.18, 157.40, 162.12, 163.84, 165.82 ppm.	
